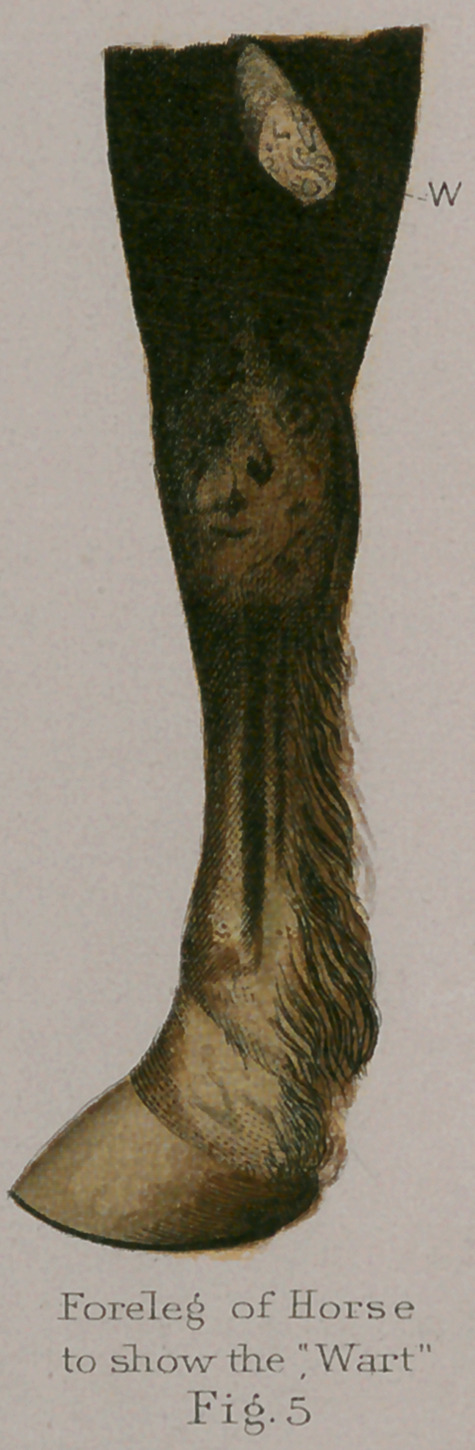# Tape Worms in Birds

**Published:** 1887-01

**Authors:** Joseph Leidy

**Affiliations:** Prof. of Anatomy, University of Pennsylvania


### THE JOURNAL

OF

### COMPARATIVE MEDICINE AND SURGERY.

VOL. VIII. JANUARY, 1887. No. 1.

5-64

### ORIGINAL COMMUNICATIONS.

### ART. I.—TAPE-WORMS IN BIRDS.

BY PROF. JOSEPH LEIDY, M. D., LL. D.

*Prof. of Anatomy, University of Pennsylvania.*

Birds are as much infested with intestinal worms as other classes of animals, and none appear to be exempt, no matter what may be the nature of their food, though aquatic birds, appear to harbor a greater number of species, as exemplified by ducks and geese. Among the parasites, tape-worms, mostly of the genus tænia, are common, though less frequent than the thread worms. The domestic fowl in Europe has been reported to harbor half a dozen different species of tænia, though I have as yet observed but one with us, and this but rarely. No species I believe has been noticed in our turkey, nor the guinea fowl and pea fowl.

FIG. 1.—Anterior extremity of Tænia microps. 30 diameters.

Dr. J. Van A. Carter, of Fort Bridger, Wyoming, directed my attention to the sage fowl, *Centrocercus urophasianus*, as being much infested with tape-worms. They often occur together in large numbers, sometimes so as to distend the small intestine. The young birds especially are affected; and the old birds appear comparatively exempt. Perhaps this may be due to the individuals much infested being killed off, though the living birds infested, which were observed, appeared not to be suffering in nutritive condition. The species seems to be the *Tænia microps*, Diesing; the same which infests the Capercailzie, *Tetrao urogallus*, of Europe. Its characters are as follows. Head globose or oval, without rostellum or armature, and with a central fovea at the vertex. The four bothria spherical or oval. Neck long, variably narrower than or as wide as the head. Anterior segments, where distinct, about three times the breadth of the length; subsequently seven or eight times the breadth of the length; afterwards again about three times the breadth of the length; then nearly square; next longer than broad; and finally two or three times the length of the breadth. Segments mostly flat, or narrowly elliptical in section, but finally nearly as thick as wide so as to be oval or nearly circular in section. Genital apertures marginal, alternating, most distinct in the middle segments. Ova, in the terminal segments, oval, colorless, with an embryo provided with three pairs of spines.

Length, 9 inches; greatest width, 2¼ lines. Measurements of several individuals were as follows:

No. 1. Length, 28 centimeters. Head, 0.375 m.m. long, 0.45 broad. Bothria, 0.225 by 0.25. Neck, 2 m.m. long; 0.25 where narrowest. Anterior segments, 0.125 to 0.175 long, 0.75 to 1 m.m. broad; middle segments, 1.5 long, 4.5 broad; terminal segments, 2 m.m. long, 1 broad and thick.

No. 2. Length, 11 centimeters. Terminal segments, 1 m.m. long, 4 broad.

No. 3. Length, 17 c.m. Head as in No. 1. Neck, 2.5 m.m. long. Anterior segments, 0.125 m.m. long, 0.3 broad and then 0.45 broad. One-third the length behind, 1 m.m. long, 2.25 broad; terminal segments, 3 long by 1 to 1.25 broad.

No. 4. Length, 15 c.m. Terminal segments, 1.5 m.m. long, 4.5 broad.

No. 5. Length, 22 c.m. Terminal segments, 1.5 m.m. long, 4 broad.

No. 6. Length, 16 c.m. Middle segments, 1 long, 2.5 broad; terminal segments, 2 long, 1.5 broad, 1 thick.

No. 7. Length, 25 c.m. Middle segments, 1 m.m. long, 5 broad; at posterior fourth, 2 long, 3 broad; terminal segments, 3 long, 2 broad, 1.5 thick.

No. 8. Terminal segments, 5 m.m. long, 1.5 wide, 1 thick.

No. 9. Terminal segments, 3.3 m.m. long, 2 wide and thick. Ova, 0.12 m.m. long, 0.08 broad.

Our reed bird, or rice bird, *Dolichonyx oryzivorous*, at the time of its autumnal visit to the vicinity of Philadelphia, I have found to be very much infested with tape worms. Every bunch of a dozen, as obtained in market, will be found to have

*Fig. 2.*

*Fig. 3.*

*Fig. 4.*

FIGS. 2, 3, 4.—Tænia pestifera. 2, head; 3, anterior segments; 4, posterior segments. 40 diameters.

three or four individuals with the parasite. The worms usually are found in the thin birds, while the fat ones are commonly free; thus apparently indicating by their presence an influence on the nutrition of their host. The species formerly described by me, and since more carefully examined, presents the following characters:

*Tænia pestifera.*—Proc. Acad. Nat. Sci. 1855, 443. Head quadrate, scarcely defined from the neck, summit truncate, slightly prominent, flat, or depressed, unarmed; bothria large, spherical, occupying the four corners. Neck long, as wide or slightly narrowed from the head. Body gradually widening to the middle and then more or less tapering; anterior segments transversely linear, becoming gradually longer and broader; subsequently obcuneate longer and narrower.

In an apparently complete individual, 3½ inches long, it was widest at the middle and tapering towards the extremities.

Head, 0.75 m.m. broad; middle segments of body 1.5 broad; posterior segments, 0.75 broad.

In a number of individuals the head ranged from 0.25 m.m. long and 0.875 broad to 0.3 long by 1.625 broad. Posterior cuneate segments variably 0.3 to 0.375 m.m. long by 0.5 to 0.75 wide.

I have seen several tape-worms, apparently of the same species, submitted to my examination by Dr. B H. Warren, of West Chester, who obtained them from the yellow-breasted chat, *Icteria virens.*

Prof. S. F. Baird, submitted to my examination a number of tape-worms obtained from the cow bird, *Molothrus ater*, which I at first supposed to be of the same species as the former, but comparison proves them to be different. Their characters are as follows:

*Tænia urnigera.*=*T. pestifera* in part, Proc. Acad. Nat. Sci. 1855, 443.

*Fig.* 5. *Fig.* 6. *Fig.* 7. *Fig.* 8.

FIGS. 5, 6, 7, 8.—Tænia urnigera; 5, anterior part of body; 6, 7, 8, segments from anterior, middle and posterior parts. 40 diameters.

Head urniform or cruciform, summit projecting in a pedicillate rounded knob or disk, or rostellum, unarmed. Bothria spherical, prominent. Neck, short or longer, obconic. Body, narrowest at commencement and gradually widening to near the posterior extremity. Anterior segments narrow, annular, soon becoming cuneate, gradually longer and wider, and then campanulate with prominent back border. Length from 1 to 2 inches, ordinarily about 15 lines; greatest width 0.75 m.m. Head, 0.4 m.m. broad, with neck 0.5 long; commencement of body 0.15 wide; anterior segments 0.1 long, 0.15 wide; later, 0.2 long, 0.375 wide; subsequently 0.25 to 0.75 long and 0.625 wide above and 0.875 at posterior border; a terminal segment 1.375 long, 0.5 wide.

Dr. B. H. Warren, of West Chester, a zealous orinthological observer, has submitted to my examination a collection of intestinal worms, recently obtained by him during an expedition to Florida. Among these are a number of tape-worms, of which most appear to be undescribed species. They are as follows:

*Tænia odiosa.*—Head hemiovoid to conical, unarmed; bothria

*Fig.* 9.

*Fig.* 10.

*Fig.* 11.

FIGS. 9, 10, 11.—Tænia odiosa; 9, anterior part of body; 10, 11, annulated segments from middle and posterior parts of body. 20 diameters.

subterminal, spherical, near together; neck none; body immediately after as wide or nearly as wide as the head; anterior segments short, linear; succeeding segments all wrinkled annularly, the more anterior band-like, the posterior barrel shaped. Generative apertures lateral, mostly not visible. Length, 1½ to 2 inches. Head, 0.3 to 0.45 m.m. wide; body just behind about as wide as the head; anterior segments 0.05 long; succeeding segments 0.15 long by 1 to 1.25 wide; at widest part of body, 0.5 long by 1.625 wide; posterior segments, 1.25 long by 1.25 wide. From the intestine of the quail, *Ortyx virginianus,* four birds of the same brood.

*Tænia viator.*—Elongate clavate, broadest behind and rounded at the extremity. Head longitudinally oval or cylindroid, with large, prominent spherical bothria, and with a protrusil, cylindrical proboscis, ending in a disk, but unarmed. Neck short, obconic. Commencement of the body narrowest; early segments transverse linear, the succeeding ones becoming

*Fig.* 12. *Fig.* 13. *Fig.* 14.

Figs. 12, 13, 14.—Tænia viator; 12, anterior part of body; 13, 14, segments posteriorly. 40 diameters.

longer, wider and obcuneate or subcampanulate to campanulate, with thickened posterior margin. From 6 to 18 lines long by 1.5 m.m. where widest. From the intestine of the swallow-tailed kite, *Elanoides forficatus.*

Many specimens from two birds. The worms have a yellow color; those from one bird of a bright lemon yellow, and on pressure exuding a bright deep yellow oil.

Head with proboscis, 0.7 m.m. long; breadth 0.425; length of proboscis, 0.2 to 0.3 long, 0.075 wide at middle and 0.125 at terminal disk. Width at commencement of body, 0.2 to 0.225; anterior subcampanulate segments 0.175 long and 0.3 wide; others 0.15 long and 0.45 wide; posterior larger segments, 0.375 to 0.625 long and 1.25 to 1.5 wide. In a specimen 15 lines long after some of the subcampanulate segments

0.15 long and 0.45 wide followed others much elongated 0.625 long by 0.15 wide in front and 0.25 wide behind. In the same specimen the posterior broadest segments were 0.625 long by 1.5 wide; and the last few segments were 0.75 long by 0.875 wide. In the largest segments the genital apertures could be distinguished in one margin; but neither ova nor penis could be detected.

*Tænia vexata.*—Head armed, transversely quadrate oval, with spherical bothria, variably prominent or retracted, summit convex or depressed, with a broad immersed corona of minute nooks. Neck short or sometimes longer and proportionately narrower; segments of body varying from linear to crateriform and campanulate; the last segment half oval; genital apertures marginal, mostly not readily detected. Length from 9 lines and 1 inch to 2½ inches. Head 0.375 m.m. broad; neck 0.3 broad; widest part of body 1. to 1.25

*Fig.* 16.

*Fig.* 15. *Fig.* 17.

FIGS. 15, 16, 17.—Tænia vexata; 15, anterior part of body; 16, 17, segments from more posterior part of body. 40 diameters.

m.m. broad. Intestine of pileated woodpecker, *Hylotomus pileatus.*

From six birds. In different specimens the measurements were as follows: Head, 0.325 to 0.5 m.m. wide; neck, 0.125 to 0.3 wide; anterior segments, linear, 0.0725 long by 0.25 wide; succeeding, reversed cup-like and variable in proportion of breadth and length according to degree of contraction, 0.175 long by 0.3 wide, or 0.2 long by 0.375 wide, or 0.25 long by 0.75 wide: widest segments reversed crateriform, 0.5 long by 1 wide, or 0.3 long by 1.25 wide, or 0.25 long by 1.5 wide; posterior segments campanulate, 0.375 long by 0.625 wide, or 0.5 long by 1. wide, or 0.75 long by 1. wide.

In a much contracted specimen 1½ inches long, with the widest part 1.75 m.m. broad and proportionately thickened, the head was 0.4 wide, the neck, 0.25, the anterior cup-like segments 0.15 long by 0.2 wide; the posterior widest and thickest part with segments, 0.25 long by 1.75 wide; the posterior companulate segments, 0.75 long by 1. wide. In another much contracted specimen 30 m.m. long, the head was 0.325 m.m. wide; the short neck, 0.225 wide; the widest part with segments 0.3 long and 1.625 wide; the terminal segments, 0.375 long by 1.25 wide. In a much elongated specimen of 2½ inches, the head was 0.375 m.m. wide; the neck, 0.2 wide, the anterior cup-like segments, 0.2 long and 0.375 wide; succeeding segments, 0.25 long and 0.75 wide; next 0.75 long by 1. wide; and terminal ones 0.5 long by 0.8 wide.

In some fragments with broad segments the genital apertures were seen to be distinctly marginal and with a small penis having the summit protruding.

*Tænia simpla.*—Head short, transverse discoid or hemispherical, slightly prominent or depressed at summit, unarmed; bothria spherical, occupying the four corners; neck, none; body at commencement variably narrower than the head;

*Fig.* 18.

FIG. 18.—Tænia simpla; anterior part of body. 40 diameters.

anterior segments linear, becoming gradually longer and broader and decidedly campanulate. Many fragments, from which the worm is estimated at about 18 lines. Intestine of the chuck-will-widow, *Antrostomus carolinensis.* Head, 0.2 m.m. wide, 0.1 long. Body succeeding head, 0.175 m.m. wide. Campanulate segments, 0.375 long by 0.5 wide; subsequently same length and double width. Some of widest segments, 0.25 m.m. long by 1.25 wide.

*Tænia aurita,* Rudolphi.—Head urn-like or doubly conical, summit armed with a double circle of alternating hooks, long and short; bothria lateral, spheroidal; neck obconic; body long, clavate, widening behind and abruptly rounded at the end; anterior segments linear, then linear campanulate, and then more strikingly campanulate with the posterior border thickened. Length to about 2 inches. Head and neck 0.3

m.m. long, 0.2 wide; narrowest portion of body 0.06 to 0.1 m.m. wide; near posterior extremity to 1. m.m. wide. In a small and apparently complete individual of 10 lines, the head was 0.225 m.m long; commencement of the body 0.1 m.m. wide; three lines behind the head the segments were 0.075 m.m. long by 0.25 wide; near the middle 0.15 long

*Fig.* 19.

*Fig.* 20.

*Fig.* 21.

*Fig.* 22.

Figs. 19, 20, 21, 22.—Tænia aurita; 19, head, 125 diameters; 20, hook, 500 diameters; 21, 22, segments near middle of body, 40 diameters.

and 0.75 wide. Terminal segments 0.2 long by 0.825 wide to 0.25 long by 0.75 wide. A posterior fragment of 15 lines had the anterior campanulate segments 0.125 m.m. long by 0.5 wide and the posterior segments 0.35 m.m. long by 1 wide. Bothria 0.08 m.m diameter. Long hooks 0.048 long; short ones 0.028.

*Tænia unilateralis*, Rudolphi.—From the intestine of the blue heron, *Florida coerulea.* Previously described from the same bird from Brazil. Head very small, consisting of four small bothria terminating the body, with a minute nail-like rostellum. Neck none. Body rapidly widening from the head. Anterior segments linear; posterior segments short cuneate, with a minute cylindrical penis projecting from each segment, all on the same side. From the intestine of the green heron, *Butorides virescens.* Many fragments. In the only one with the head, 2 inches in length, the head was 0.3 m.m. wide; the rostellum 0.15 long; the widest part of the body 3 m.m.

*Tænia oprornis.*—Head hemispherical; bothria spherical, occupying the sides of the former; neck none; body at commencement as wide as the head and thence gradually widening to the posterior third and then diminishing. Anterior

*Fig.* 23.

*Fig.* 24.

*Fig.* 25.

FIGS. 23, 24, 25. Tænia oprornis; 23, anterior part of body; 24, 25, segments posteriorly. 40 diameters.

segments linear and transversely deeply striate; subsequently reversed dish-shaped and finally reversed bowl-shaped. Length about 18 lines. Head 0.375 m.m. to 0.45 broad; widest segments 0.125 long by 0.75 wide; posterior segments 0 2 by 0.625 wide. Several specimens and fragments from the intestine of the Kentucky warbler, *Oporornis formosa.*

A tape-worm from the robin, *Turdus migratorius,* obtained at West Chester by Dr. Warren, seems to be the same as the *Tænia angulata,* Rudolphi, infesting European species of thrushes. Its characters are as follow:

Head broader than long, with spherical bothria at the lateral angles; a short conical rostellum enclosing a conical papilla closely covered with two rows of alternating recurved hooks. Neck none. Body at commencement nearly as wide

*Fig.* 26.

*Fig.* 27.

FIGS. 26, 27.—Tænia angulata; 26, anterior part of body; 27, posterior segments. 40 diameters.

or as wide as the neck, with linear segments; subsequently obcuneate becoming gradually wider; the widest subcuneate

A fungating sebaceous Cyst.
in Parotid region.
Fig. 2.

Arm of Lemur Catta at birth,
showing the gland described
in text.
Fig. 4

Hand of Hapalemur griseus
to show the spiny patch A.
Fig. 3.

The Uropygial gland
of a Hornbill in section.
Fig. 1.

Foreleg of Horse
to show the "Wart"
Fig. 5

A. Hoen & Co. Lithocaustic, Baltimore.

twelve times as broad as long. Estimated to be about 2 inches in length, with the greatest breadth about 3 m.m.

In addition to the foregoing, I have observed *Tænia variabilis*, Rud., in our woodcock, *Philohela minor*, previously described from various European wading birds; and *Tænia scolopendra*, Diesing, in the horned grebe, *Podiceps cornutus*, previously described from a Brazilian grebe.

## Figures and Tables

**Fig. 1. f1:**
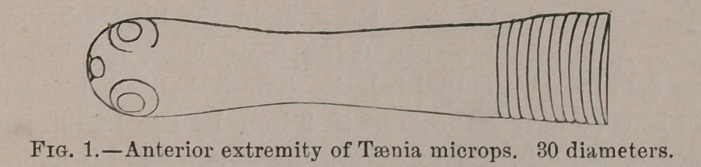


**Fig. 2. Fig. 3. Fig. 4. f2:**
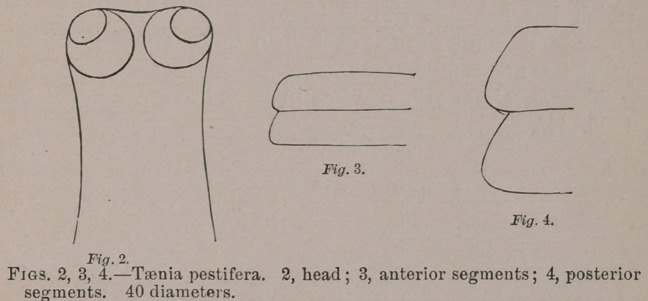


**Fig. 5. Fig. 6. Fig. 7. Fig. 8. f3:**
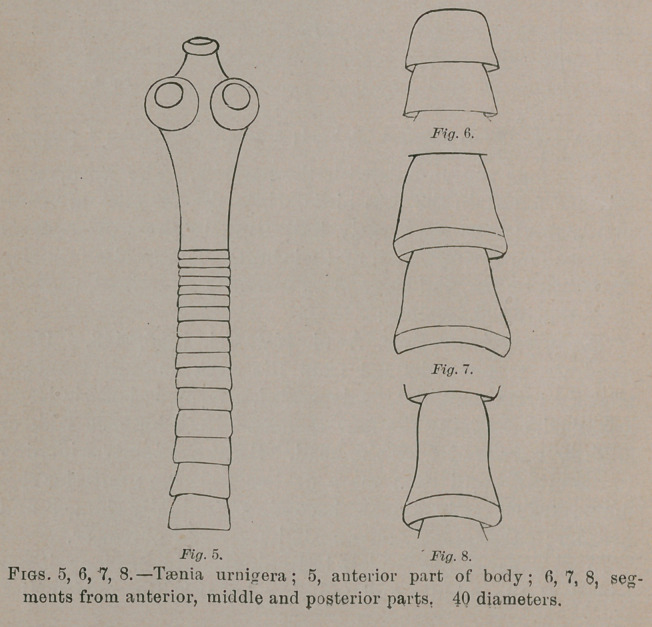


**Fig. 9. Fig. 10. Fig. 11. f4:**
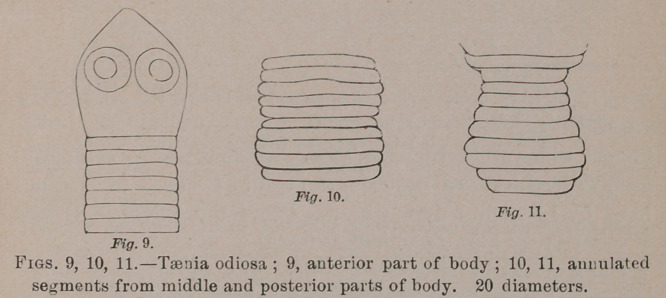


**Fig. 12. Fig. 13. Fig. 14. f5:**
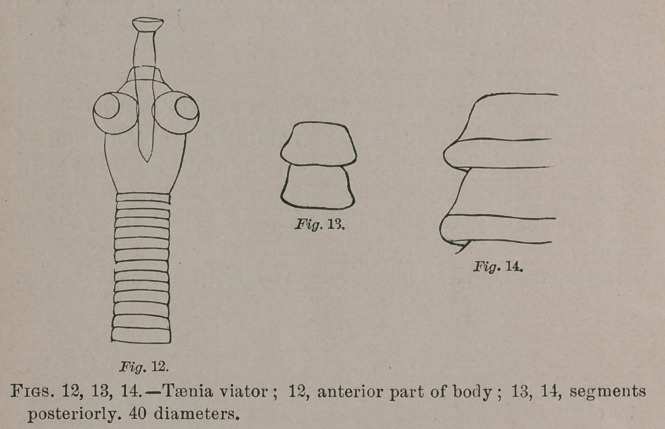


**Fig. 15. Fig. 16. Fig. 17. f6:**
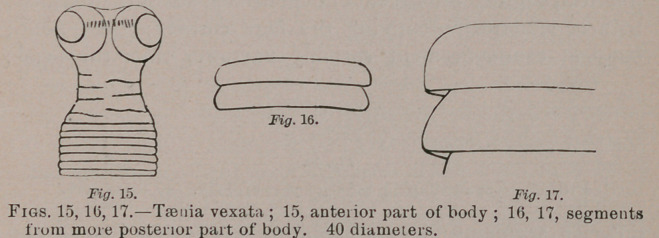


**Fig. 18. f7:**
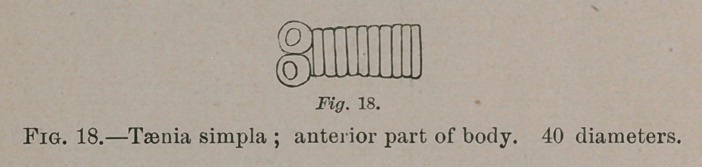


**Fig. 19. Fig. 20. Fig. 21. Fig. 22. f8:**
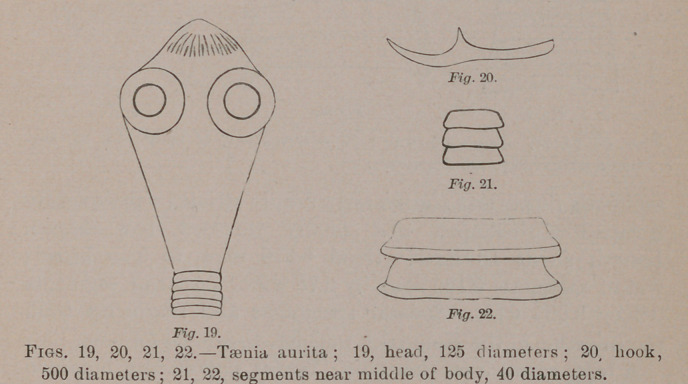


**Fig. 23. Fig. 24. Fig. 25. f9:**
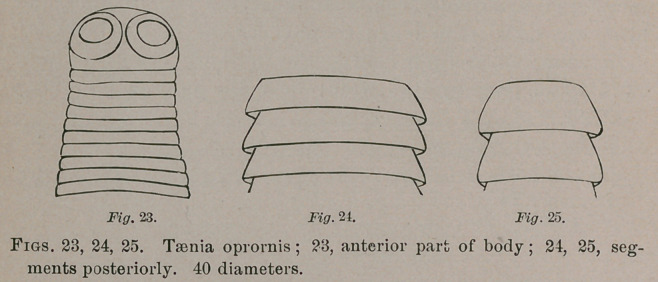


**Fig. 26. Fig. 27. f10:**
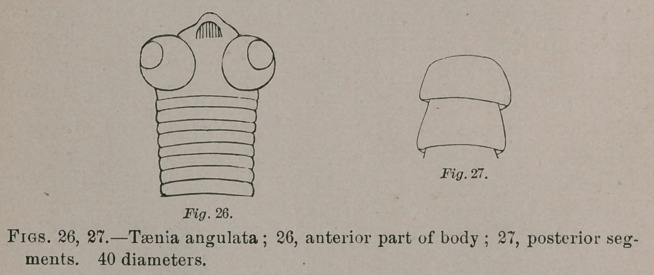


**Fig.1. f11:**
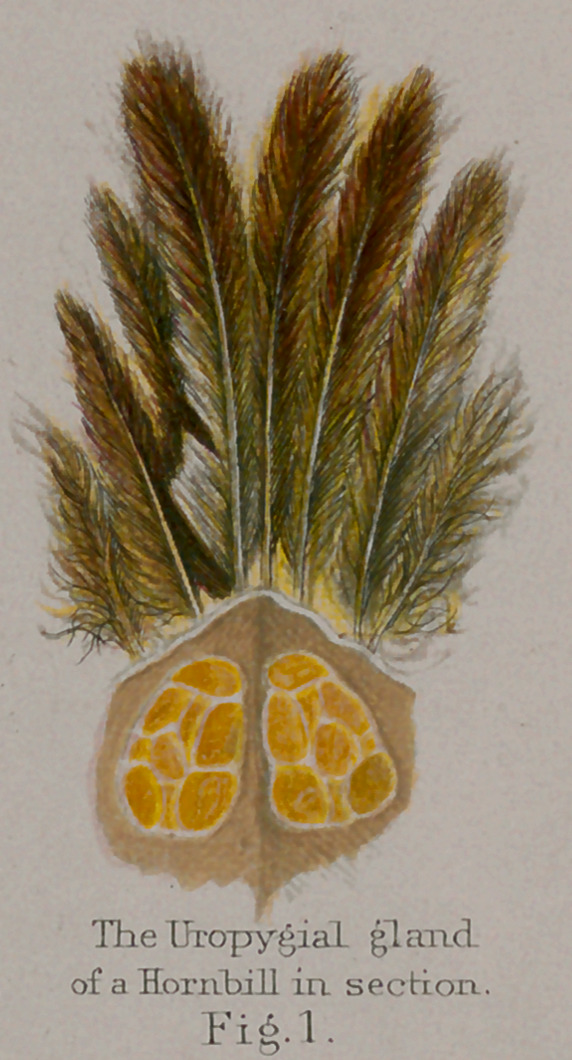


**Fig.2. f12:**
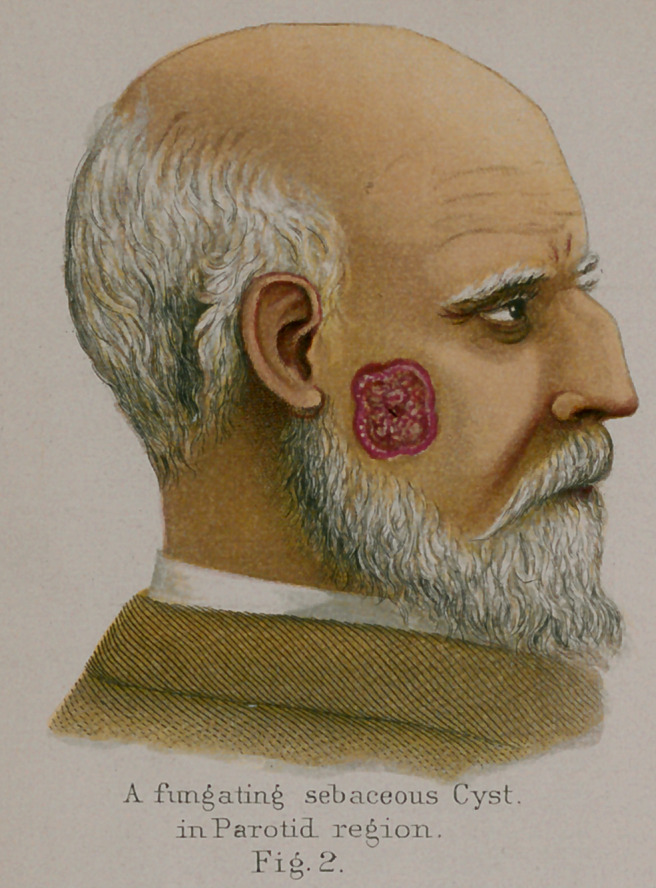


**Fig.3. f13:**
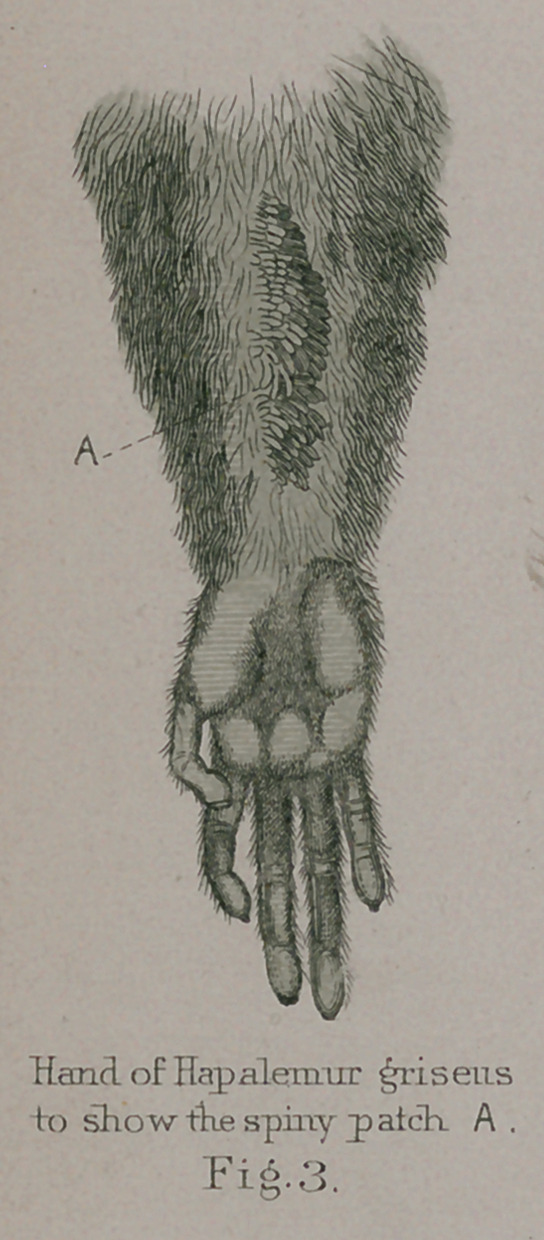


**Fig.4 f14:**
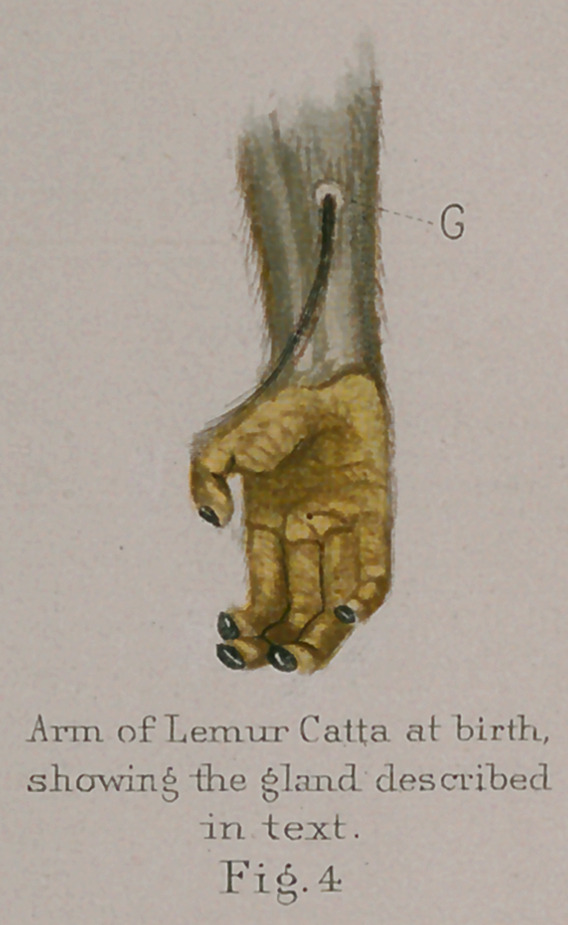


**Fig.5 f15:**